# Supplementary material for: Validating the Perceived Active School Travel Enablers and Barriers–Parent (PASTEB–P) Questionnaire to Support Intervention Programming and Research
Source: Int J Environ Res Public Health. 2023 May 19;20(10):5874. doi: 10.3390/ijerph20105874 (PMC10218046; doi:10.3390/ijerph20105874)
Supplement: Supplementary file 1 [file ijerph-20-05874-s001.zip › ijerph-2264433-supplementary.pdf]

Supplemental Materials

CFA Structural Model for Perceived AST Barrier Constructs

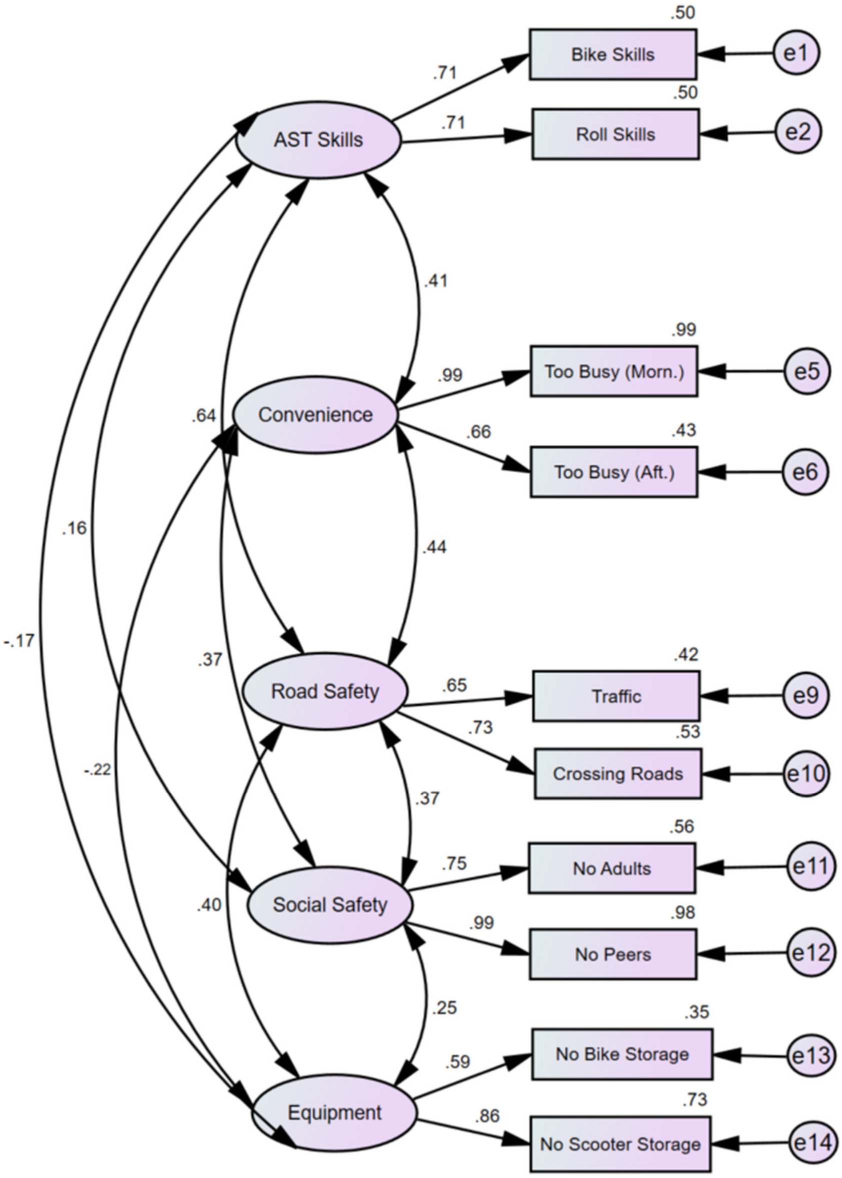

Figure A1. Confirmatory factor analysis: the figure extracted from AMOS shows the results of the covariances and factor loadings.

CFA Structural Model for Perceived AST Enabler Constructs

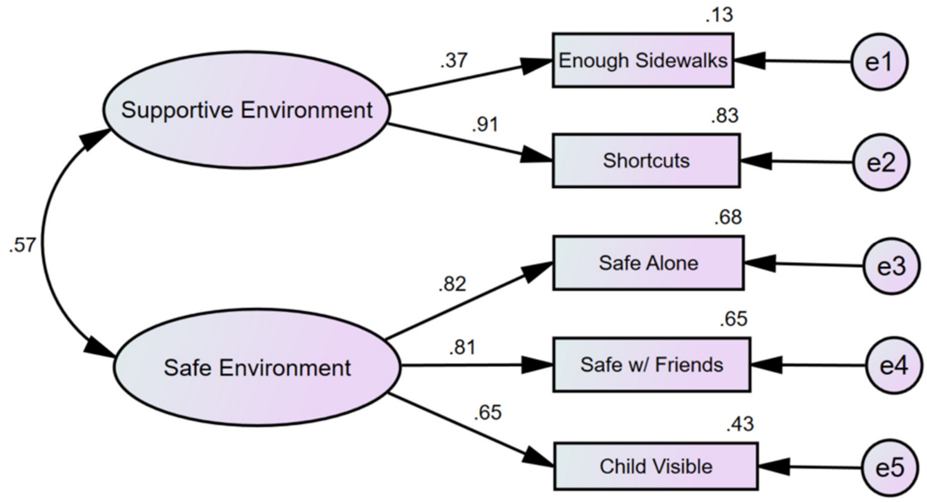

Figure A2. Confirmatory factor analysis: the figure extracted from AMOS shows the results of the covariances and factor loadings.

The 15-Item PASTEB-P questionnaire

Construct	Question	Scale			
		Strongly Agree	Agree	Disagree	Strongly Disagree
	**Perceived AST Barriers**				
AST Skills	My child does not have the skills to bike.	○	○	○	○
	My child does not have the skills to roll.	○	○	○	○
Convenience	We do not have enough time in the morning.	○	○	○	○
	We do not have enough time in the afternoon.	○	○	○	○
Road Safety	Too much traffic on the route.	○	○	○	○
	A child must cross busy roads.	○	○	○	○
Social Safety	No adults/high school students to walk with.	○	○	○	○
	No peers (e.g., friends and siblings at the school) to walk with.	○	○	○	○
Equipment Storage	Nowhere for my child to safely leave their bike at school.	○	○	○	○
	Nowhere for my child to safely leave their scooter at school.	○	○	○	○
	**Perceived AST Enablers**				
Supportive Environment	There are enough sidewalks along the route between home and school.	○	○	○	○
	There are walking paths and cut-throughs to shorten the route between home and school.	○	○	○	○
Safe Environment	My neighbourhood is safe enough for children to walk/bike/roll to and from school alone.	○	○	○	○
	My neighbourhood is safe enough for children to walk/bike/roll to and from school with friends.	○	○	○	○
	My children are visible to my neighbours when walking, biking, and rolling along their route to and from school.	○	○	○	○
